# Older Adults’ Perceived Barriers to Participation in a Falls Prevention Strategy

**DOI:** 10.3390/jpm11060450

**Published:** 2021-05-23

**Authors:** Júlio Belo Fernandes, Sónia Belo Fernandes, Ana Silva Almeida, Diana Alves Vareta, Carol A. Miller

**Affiliations:** 1 Department of Nursing, Escola Superior de Saúde Egas Moniz/PaMNEC—CiiEM, Almada, Rua António José Batista n°116, 3esq., 2910-397 Setúbal, Portugal; 2 Department of Nursing, Projetar Enfermagem, 1600-577 Lisboa, Portugal; 3 Department of Nursing, Centro Hospitalar de Setúbal, 2910-397 Setúbal, Portugal; anasilvalmeida@gmail.com (A.S.A.); diana_vareta@hotmail.com (D.A.V.); 4 Independent Care Manager at Care & Counseling, Brecksville, OH 44141, USA; cmiller4321@ameritech.net

**Keywords:** accidental falls, fall prevention, older adults, barriers, patient compliance

## Abstract

There is a need to increase older adults’ access and adherence to falls prevention strategies. This study aims to explore older adults’ perceived barriers to participation in a fall prevention strategy. A qualitative descriptive approach was used. Semi-structured interviews were conducted with 18 older adult users of a Day Care Unit from a Private Institution of Social Solidarity in the region of Lisbon and Tagus Valley in Portugal. The recruitment was made in September 2019. The interviews were recorded transcribed verbatim and analysed thematically using the method of constant comparisons. The barriers to participation in a fall prevention strategy are healthcare system gaps, social context, economic context, health status, psychological capability, and lack of knowledge to demystify myths and misconceptions about falls. There are different barriers to participate in a fall prevention strategy. It is urgent to eliminate or reduce the effect of these barriers to increase older adults’ participation in fall prevention strategies.

## 1. Introduction

Every year, one out of three older adults falls. It is estimated that each year, more than 640,000 people die as a result of falls [1]. Falls are the second leading cause of non-fatal and fatal injuries among older adults [2,3,4]. Older adults with a high risk of falls are in the high-risk group for fall injury [5] and fall-related death [6]. Fall related injury can range from minor trauma to severe injuries requiring hospitalization. The most common severe injuries include fractured bones and soft tissue injuries [7,8].

Preventing falls and fall-related injuries is challenging because of its multifactorial nature [9]. Several studies have identified more than 400 potential risk factors for falling [10]. Results from numerous researches have suggested that multidimensional falls prevention strategies can be effective in reducing the number of falls [11,12,13]. Furthermore, several guidelines have been developed to summarise the best evidence and guide healthcare professionals in their clinical practice [14,15]. Despite these facts, many fallers do not seek any type of help to prevent further falls [16], as well as many older adults, do not engage in fall prevention strategies even after referrals are made [17].

To improve access and adherence to falls prevention strategies, health care policy-makers and health administrators should contemplate older adults’ perspectives when developing these strategies [18]. Little is known about the barriers to engage in a fall prevention strategy in the Portuguese population, therefore this research seeks to fill this evidence gap by exploring barriers to participation in a fall prevention strategy from the perspective of Portuguese older adults with a high risk of falling. By seeking this evidence we can provide insight into developing a more effective fall prevention strategy and take measures to minimise those obstacles and therefore increasing enrolment and participation.

## 2. Methods

### 2.1. Study Design

A qualitative descriptive study was conducted using semi-structured interviews, which enabled an in-depth exploration of older adults’ perceived barriers to participation in a fall prevention strategy. To ensure quality in the research report we followed the con-solidated criteria for reporting qualitative research (COREQ) [19].

### 2.2. Setting

The study setting was a Day Care Unit from a Private Institution of Social Solidarity in the region of Lisbon and Vale do Tejo in Portugal that caters to a population of over 80,000 people. 

### 2.3. Sampling and Recruitment

The study population consists of older adults who are users of the Day Care Unit. The sampling method selection was non-probabilistic by convenience. The inclusion criteria included: (1) have a high risk of falling; (2) have participated in a falls prevention strategy.

Researchers used the fall risk test developed by VeiligheidNL [20] to screen older adults. The test contains three simple questions: ‘Did you fall during the past twelve months?’, ‘Do you experience problems with movement and balance?’, and ‘Are you afraid of falling?’ When participants answer “yes” to the first question or two of the overall questions, they are considered at high risk of falling.

The recruitment was made in September 2019. Eligible participants were invited by telephone to participate in the interviews. All older adults available at the time of data collection that met the inclusion criteria were included in the study.

### 2.4. Participants

Of the 26 older adults who agreed to participate in the study, 8 were excluded based on not meeting the inclusion criteria. We conducted 18 interviews, the participants were mostly male (61.1%). The mean age of participants was 76.2 (range 69–83 years) and the standard deviation was 4.16215 years.

### 2.5. Data Collection

The interviews were conducted by the first author at the Day Care Unit facilities and lasted approximately 20 min. All interviews were audiotaped, transcribed verbatim into written data, anonymised, and analysed.

The semi-structured interview guide was developed based on data gathered from previous studies and with contributions from experts. Examples of questions used in the guide are: ‘Were there any factors that limit you to participate in a falls prevention program?’ ‘Tell me about a particular example of a barrier to undertake a falls prevention program?’ ‘What do you think could difficult people to participate in a falls prevention program?’ ‘Do you have any suggestions for how services could improve its contributions for older adults to engage in a falls prevention program?’

### 2.6. Data Analyses

In the process of analysis, Braun, Clarke, Hayfield, and Terry’s [21] procedures were followed. Researchers listened to the audio records to obtain an overall sense and then transcribed verbatim into written data. The data was separated into meaning units, based on similarity. Meaning unit codes were developed based on participants’ own words. Initially, the transcribed verbatim was reviewed independently by two study team members and manually coded using inductive content analysis to identify common themes. To ensure credibility, the researchers discussed and compared the emergent themes and categories. Afterward, the other study team member reviewed the participant quotes and matched each quote to one of the identified themes.

### 2.7. Ethics and Procedures

Before conducting the study, a research protocol was analysed and approved by the Institutional Review Board. Prior to the interviews, all participants sign a written informed consent to record, anonymously report and publish the research data.

## 3. Results

Six themes emerged from the analysis of focus group data. These themes included several categories as showed in Table 1, and examples are provided in the following section.

### 3.1. Healthcare System Gaps

#### 3.1.1. Access Shortage

Participants highlighted a shortage of program offers. Hence, they were unable to engage in a fall prevention program.

‘There are not many offers of these types of programs. While I was in the hospital, I participated in an exercise program to prevent falls, but after discharged I questioned everyone and nobody was able to refer me to a community-based program. There is a real shortage.’

(P11)

#### 3.1.2. Lack of Personalised Interventions

In addition to the offer shortage, participants reported that the ones they attended lack personalised interventions. They felt that to solve their problem, they should have been targets of tailored programs. Program participants had different problems, so they should receive interventions personalised to their different needs.

‘There’s no personalised intervention. There were around ten or twelve people in a room doing the same thing. My problem was not the same as the others, so how does the same intervention solve different problems.’

(P3)

### 3.2. Social Context

#### 3.2.1. Stigma Associated with Fall

Participants described stigma as a barrier to engage in a fall prevention strategy because they associate falls with the need to receive institutional care. They fear that by participating in a falls prevention strategy, their families might assume they lack the physical capacity to take care of themselves and therefore institutionalize them to nursing homes.

‘It is unlikely for me to assume the need to undertake a fall prevention program because as soon as they feel that I lack capabilities to take care of me, they will search for a nursing home.’

(P4)

#### 3.2.2. Social Awkwardness

The group environment made participants state social awkwardness as they felt uncomfortable and unease in a group-based exercise program.

‘At this age, it isn’t normal for us to do training exercises in a gym. You look around and realise everybody feels uncomfortable. Life is full of awkward and uncomfortable situations. We can avoid them. You have a lot of say-so in how you feel as you grow older.’

(P8)

### 3.3. Economic Context

#### Financial Capacity

Participants referred that their financial capacity is a major barrier to undertaking a falls prevention strategy. Even considering the offer of free programs, in some cases, the costs associated with travel in itself harm their family budget.

‘Even if there was a greater offer of fall prevention programs, they are not free and you have to add travel costs. As you grow older, the money you spend on our health increases a lot.’

(P10)

### 3.4. Health Status

#### 3.4.1. Lack of Physical Fitness

Participants reported their experience of facing physical challenges. They consider that despite thinking that they were able to perform the physical component of the fall prevention program, they lacked physical fitness.

‘It was a bit more than I could manage. I thought I was capable to perform the exercises without any problems, but the reality was different. I felt short of breath and tired. I went there once and gave up.’

(P7)

#### 3.4.2. Impaired Mobility

Besides their lack of physical fitness, participants also reported their inability to face physical challenges due to impaired mobility. This impairment has an impact not only on the ability to perform exercises but also on the ability to travel from home to the facilities where the training program takes place.

‘There were several problems. First, my impaired mobility has a tremendous impact on my ability to travel to the clinic. Then I was unable to do more than half of the exercise because of my mobility.’

(P2)

### 3.5. Psychological Capability

#### Fear of Falling and Injuries

Participants referred to the fear of falling and injuries as a barrier. They stated that after several falls, even if they did not sustain any injuries, they become afraid of falling and this leads them to feel more and more limited in terms of their autonomy and physical independence.

‘Who wants to participate in any exercise program if they have to leave their house and risk falling again. It’s a big problem because you fall, and fall again and then you became afraid. Fear sets in and you don’t want to do any daily activities.’

(P12)

### 3.6. Lack of Knowledge

#### 3.6.1. Falls Perceived as Inevitable and Not Preventable

Participants referred that their lack of knowledge about falls led them to think that this was an issue that only affected frail older adults and that falls are accidental and, therefore, inevitable and not preventable.

‘There is a belief that falls only happen to frail older adults. I thought they were an inevitable event associated with ageing. It wasn’t just me who thought that. My sons also thought the same. Lack of knowledge leads to these common misconceptions.’

(P12)

#### 3.6.2. Underestimation of Risk

Participants reported that some older adults refused to admit that they are at risk of falling and thus underestimate the consequences of certain behaviours to increase the risk of fall.

‘Sometimes people do not want to admit their weakness and they are careless, underestimate the consequences of their behaviours and then fall and sustain injuries.’

(P1)

## 4. Discussion

Understanding the barriers to undertaking a falls prevention strategy may influence the guideline to change the process of selecting and more appropriately target the person at risk of falling.

It is well known from information gathered in other researches that before choosing the program interventions, decision-makers need to understand the target group, setting, and barriers to change [18].

This research found that access shortage and lack of personalised interventions are healthcare system gaps that could act as barriers for older adults to undertake a falls prevention strategy. Participants described difficulties with accessing any type of fall prevention programs due to access shortage. Older adults require opportunities within their environment to attend these types of programs. There must be a variety of offers so that the person can choose a program that best suits one’s personhood [22]. 

It seems clear, that for the successful implementation of a fall prevention strategy in community settings, it requires an approach involving a greater program offers. Given the limited resources in Portugal, it is likely that potential users will become unmotivated after realising that the resources are scarce and scattered.

A theme articulated amongst participants was the lack of personalised interventions. The identification of this barrier may reflect the value attributed by participants in maintaining their personhood and usual routines. Further, previous studies have shown that older adults may value the affective characteristics of care as much as achieving better health outcomes [23]. It has been known for a long time that intervention strategies should be tailored to the cultural and socioeconomic context. There are no strategies that have universal applicability [24]. There is a growing body of evidence demonstrating that personalised interventions should be applied to each person to achieve positive clinical outcomes and increase their satisfaction [25]. This type of care can be the path to include the person at the centre of care and improve older adults’ adherence to falls prevention strategies. Despite this acknowledgment, reports from participants revealed that in the programs they engaged in, this did not happen.

Another barrier highlighted in this study is the social context, namely the stigma associated with falls and social awkwardness. According to participants, older adults perceived falling as a stigma as they related falls with declining capabilities and loss of independence and consequently with the need to be admitted in a residential or nursing home. Many older adults feel embarrassed and stigmatised about their falls, and consequently choose not to show their weakness [26]. 

Similar findings have been identified among older adults in Eastern Culture. In older Chinese people, there was a refusal to use walking aids as they perceived them as a bad omen and carried stigma [27].

It is necessary to remove the stigma associated with falling so that older adults can get the help they need to promote healthy and active aging. Therefore, fall prevention strategies should convey the message of positive health and social benefits, such as improving muscle strength and body balance rather than focusing on reducing falls [28].

Participation in group programs can lead to social benefits and often act as an enabler for continued participation, although the transition to new groups could be challenging [29]. In this study, the participants considered the group environment as socially awkward because they felt out of place in that environment. This barrier can be linked closely to an individual’s preference as in other researches the group environment was appointed either as an enabler or a barrier to continuing with a fall prevention program [29]. 

Another identified barrier is the economic context. This barrier has a crucial preponderance for older adults’ participation in falls prevention strategies, because if they do not have the financial capacity to support their daily expenses, they will not consider undertaking any other activities. Other studies also identified the financial situation as a barrier to participate in a fall prevention strategy [30,31]. However, there seems to be a consensus in the literature that the cost associated with intervention may not be perceived as a barrier, as long as the cost is fair and reasonable enough [26]. 

Regarding the health status, participants reported their experience of facing physical challenges. Other researchers also identified the lack of physical fitness and impaired mobility as major barriers to undertake a falls prevention strategy [32,33]. In addition, they identified that previous habits and perceived value of physical activity can be an important factor to older adults’ participation in exercise programs [32]. 

To undertake a falls prevention strategy participants must change their behaviour in the same manner as a sedentary person needs to be supported and encouraged to take an exercise program [34]. Physical training must be a progressive and adaptive process to allow the body to adapt to the stress of exercise with greater fitness [33]. Apparently, in the programs attended by participants, the exercise routines were not tailored to their needs and health status, and, therefore, they were not able to perform well. As mentioned previously, fall prevention strategies must be tailored to the context and the participants in order to increase their adherence. Furthermore, fall prevention programs must be multidimensional, combining a wide range of specific interventions that go beyond physical exercise [18,33]. 

The fear of falling was perceived as a barrier that leads older adults to feel physically limited and consequently have to protect themselves against dangers, by delegating their care to others, and ultimately denying their own autonomy and physical independence. In other researches, older adults with previous falls were more prone to undertake a falls prevention strategy, and those who were afraid of falling were four times more likely to enrol in these types of strategies [18].

The underestimation of risk and falls being perceived as inevitable and not preventable are lined up with the conviction that falls are part of ageing. These findings may suggest that falls prevention strategy are not effective and emphasise the vital need that older adults have to acquire more knowledge to demystify myths and misconceptions about falls. Educational or awareness strategies must be part of a multidimensional falls preventions strategy to counteract the common misconception that falls are simply an issue for older and frail adults as a result of accidents and, therefore, not preventable [18,33].

### Limitations and Trustworthiness

Researchers carried out 18 interviews, which is considered sufficient for data saturation to occur. Data were collected by semi-structured interview during which participants described their experiences. The overall results illuminate variations in older adults’ perspectives on barriers to undertake a falls prevention strategy. 

In this study, participants’ mental and physical status were not assessed. This is a limitation as active depression and sever physical illness could potentially influence the participants’ answers. Additionally, similar to previous studies that depend on data collected from interviews, actual reports may diverge from what participants revealed due to biases such as lack of confidence in guaranteeing anonymity or protection of identity, values, or beliefs. We collected data from the reports of various participants to reduce this bias.

The study’s trustworthiness was confirmed through credibility, transferability, dependability, and confirmability as described by Nowell, Norris, White, and Moules [35]. To increase credibility researchers debated each phase of analysis. Disagreements were solved by discussion until achieving consensus. To ensure its transferability, the researchers provide descriptions with appropriate quotations so that those who seek to transfer the findings to different sittings can judge transferability. To achieve dependability, researchers detailed every phase of the decision-making process so that others can follow the research. To ensure confirmability, external observers search for inconsistencies by comparing the similarity of their perceptions with the ones from the researchers. 

## 5. Conclusions

In conclusion, this research has shown that older adults identify different key barriers to engage in a fall prevention strategy. According to participants’ narratives, we identified six categories of barriers, namely healthcare system gaps, social context, economic context, health status, psychological capability, and lack of knowledge. 

From our point of view, some barriers need urgently to be addressed for older adults to participate in a falls prevention strategy. The healthcare system gaps and social context are major barriers that highlight the critical need to develop and disseminate fall prevention strategies through public and private partnerships and social marketing.

These programs should be structured based on personalised interventions for each person, as scientific evidence shows that tailored interventions can lead to positive clinical outcomes and increase personal satisfaction.

Another barrier and perhaps the most important is the economic context because if the person does not have the financial capacity to support the fees to carry out the program, they will certainly not engage in these activities. Falls prevention strategies must be low cost because falls are a public health problem, with impact on economic costs for healthcare systems worldwide, so prevention must be a priority. Thus, fall prevention strategies should have a minimal cost for users and ultimately be supported by the healthcare systems. 

Further research is needed to better understand the relationships and impact of these barriers, also it would be valuable to study which barriers are the drives of success to participation in a falls prevention strategy.

## Figures and Tables

**Table 1:** Barriers to undertaking a fall prevention strategy.

Themes	Categories	Participants (*N* = 18)
Healthcare system gaps	Access shortage	12
	Lack of personalised interventions	7
Social context	Stigma associated with fall	8
	Social awkwardness	7
Economic context	Financial capacity	12
Health status	Lack of physical fitness	7
	Impaired mobility	5
Psychological capability	Fear of falling and injuries	9
Lack of knowledge	Falls perceived as inevitable and not preventable	4
	Underestimation of risk	4

## Data Availability

The data presented in this study are available on request from the corresponding author. The data are not publicly available due to participants´privacy.
